# Cell-to-Cell Communication by Host-Released Extracellular Vesicles in the Gut: Implications in Health and Disease

**DOI:** 10.3390/ijms22042213

**Published:** 2021-02-23

**Authors:** Natalia Diaz-Garrido, Cecilia Cordero, Yenifer Olivo-Martinez, Josefa Badia, Laura Baldomà

**Affiliations:** 1 Secció de Bioquímica i Biología Molecular, Departament de Bioquímica i Fisiologia, Facultat de Farmàcia i Ciències de l’Alimentació, Universitat de Barcelona, 08028 Barcelona, Spain; ndiazgarrido@ub.edu (N.D.-G.); ccalday@gmail.com (C.C.); yeni_olivo@hotmail.com (Y.O.-M.); josefabadia@ub.edu (J.B.); 2 Institut de Biomedicina de la Universitat de Barcelona (IBUB), Institut de Recerca Sant Joan de Déu (IRSJD), 08950 Barcelona, Spain

**Keywords:** extracellular vesicles, exosomes, gut communication, gut immunity, intestinal homeostasis, miRNAs

## Abstract

Communication between cells is crucial to preserve body homeostasis and health. Tightly controlled intercellular dialog is particularly relevant in the gut, where cells of the intestinal mucosa are constantly exposed to millions of microbes that have great impact on intestinal homeostasis by controlling barrier and immune functions. Recent knowledge involves extracellular vesicles (EVs) as mediators of such communication by transferring messenger bioactive molecules including proteins, lipids, and miRNAs between cells and tissues. The specific functions of EVs principally depend on the internal cargo, which upon delivery to target cells trigger signal events that modulate cellular functions. The vesicular cargo is greatly influenced by genetic, pathological, and environmental factors. This finding provides the basis for investigating potential clinical applications of EVs as therapeutic targets or diagnostic biomarkers. Here, we review current knowledge on the biogenesis and cargo composition of EVs in general terms. We then focus the attention to EVs released by cells of the intestinal mucosa and their impact on intestinal homeostasis in health and disease. We specifically highlight their role on epithelial barrier integrity, wound healing of epithelial cells, immunity, and microbiota shaping. Microbiota-derived EVs are not reviewed here.

## 1. Introduction

Communication between cells is crucial to preserve body homeostasis and health. This is particularly relevant in the intestinal tract, where host cells are exposed to millions of bacteria and food antigens [1]. Besides cell-to-cell contacts and soluble released factors that can act locally or distantly on other cells types and tissues, extracellular vesicles (EVs) can mediate intercellular crosstalk. EVs are a heterogeneous population of lipid bilayer membrane-enclosed vesicles that transport and deliver proteins, lipids, and nucleic acids to recipient cells. There are multiple classes of EVs released from almost all cell types and they can also be found in various biological fluids. Initially, the release of EVs was considered a mechanism to discard unwanted materials from cells. However, intensive research has revealed that release of EVs has a pivotal role in cell-to-cell communication in human physiology and pathology. Here, we discuss current knowledge on the production and cargo composition of EVs and focus the attention on EVs released by cells of the intestinal mucosa, highlighting their impact on intestinal homeostasis in health and disease.

## 2. Biogenesis and Composition of EVs

### 2.1. Classification of EVs

The term EVs comprise a varied, heterogeneous group of particles released from cells that largely originate from endosomes and/or plasma membrane with complex cargoes [2]. The classification of EVs has been inconsistent and confusing. In fact, isolation methods for EV subtypes are still under development and consensus on best practices has not yet been reached. However, according to the International Society for Extracellular Vesicles (ISEV), minimal requirements must be met to assert the presence of EVs in sample isolates, and several experiments need to be conducted to characterize the existence of EVs [3]. Thereby, on the basis of current studies on the mechanism of formation, mode of release from cells, and size, we can describe three categories: (i) exosomes, (ii) microvesicles (MVs), and (iii) apoptotic bodies. Although vesicle size is the main factor used in EV categorization, some reports have demonstrated that EVs within one EV class may be heterogenic in terms of cargo and functionality.

Exosomes are small EVs defined as 30–200 nm lipid bilayer particles secreted by all cell types [4]. Recent studies have shown two novel subpopulations of exosomes, including large exosome vesicles (Exo-L) with 90–120 nm diameter, small exosomes vesicles (Exo-S) with 60–80 nm diameter, and an abundant population of non-membranous nanoparticles called exomeres with sizes around 35 nm [5]. Exosomes can be found in almost all living cells, including dendritic cells, lymphocytes, mast cells, intestinal epithelial cells, and endothelial cells. Moreover, exosomes can be detected and isolated from various body fluids such as urine, plasma, cerebrospinal fluid, human milk, and exudates [6]. Exosomes originate first as intraluminal vesicles (ILVs) within a multivesicular body (MVB), which are released into the extracellular matrix after MVB fusion with the plasma membrane. This process operates through the endosomal pathway [7]. Due to their origin, exosomes differ from other EVs that are released from the cell as the result of a direct budding process of the plasma membrane.

MVs, also called microparticles or ectosomes, are defined as 200–1000 nm vesicles released by vesiculation from eukaryotic cells [8]. They are formed by outward blebbing of the plasma membrane and subsequently released via actomyosin-driven fission of plasma membrane blebs. Initially, MVs were described as “platelet dust”, due to their identification as subcellular material originating from platelets in normal plasma and serum [9]. MVs derived from human cancer cells are called “oncosomes” (1–10 µm) and have a role in cell–cell communication. Their ability to participate in the horizontal transfer of signaling proteins and mediators contributes to invasive activity in cancer [10]. Although oncosomes are classified within MVs; this group is studied separately due to their larger size, characteristic biomolecules, and isolation methods.

Apoptotic bodies include much larger vesicles of 1–5 µm and they are formed during membrane blebbing and cellular disassembly from fragmentation when the cytoskeleton breaks at the beginning of apoptosis [11]. Their impact upon other cells is not well studied. Nonetheless, it has been suggested that apoptotic bodies participate in immune regulation, including processes such as autoimmunity, cancer, and infection [12]. This group will be excluded from this review.

### 2.2. Biogenesis of EVs

**Figure 1:**
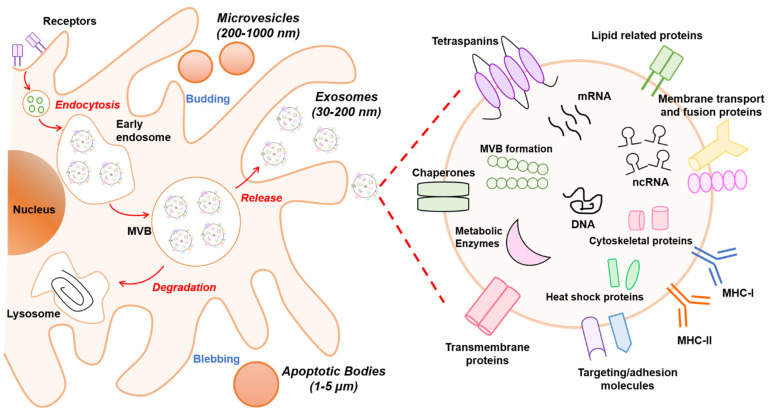
Schematic overview of extracellular vesicle (EV) biogenesis and cargo. At least three different subclasses of EVs are generated by eukaryotic cells: exosomes (30–200 nm), microvesicles (200–1000 nm), and apoptotic bodies (1–5 µm). The left panel schematically shows the biogenesis pathway for each EV type. Microvesicles and apoptotic bodies sprout directly from the plasma membrane, whereas exosomes are generated within multivesicular body (MVB) subpopulations that upon maturation fuse with the plasma membrane. The biogenesis pathway influences the cargo of EVs. In particular, the composition of exosomes is presented in the right panel. Exosomes are rich in the adhesion molecules tetraspanins (CD9, CD81, CD63), antigen-presenting molecules (MHCI/II), membrane transport proteins (annexins, flotillin), enzymes (elongation factors, metabolic enzymes), and other cytosolic proteins (ribosomal proteins). In addition, lipids (sphingomyelin and phosphatidylserine) and nucleic acids (DNA, RNA, non-coding RNAs (ncRNAs), and micro-RNAs (miRNAs)) also are bioactive components.

It is known that EVs are formed by multiple mechanisms that are summarized in Figure 1. Exosomes are generated within the endosomal system and microvesicles are formed by outward budding vesicles at the plasma membrane. Although the generation of EVs (exosomes or microvesicles) occurs at distinct sites within the cell, common intracellular mechanisms are involved in the biogenesis of both entities.

Exosomes are derived from endosomal compartments. Within the endosomal system, endosomes are divided into compartments including early endosomes, late endosomes, and recycling endosomes. To release exosomes, plasma membrane and cytosol-associated molecules (lipids, proteins, and nucleic acids) are endocytosed and transferred into early exosomes, which fuse with endocytic vesicles sorting their cargo for degradation, recycling, or secretion. The remaining early exosomes differentiate into late exosomes that give rise to ILVs formed by inward budding of the endosomal membrane. In turn, ILVs are enclosed within MVBs. These can either fuse with lysosomes if their content is destined for degradation, or merge with the cellular membrane, releasing the ILVs as exosomes into the extracellular space [13].

The exosome biogenesis pathway can be regulated by various mechanisms. One well-known mechanism involves the endosomal sorting complex required for transport (ESCRT) that is needed for MVB formation as it classifies ubiquitinated intracellular cargos that are destinated for lysosomal degradation into MVBs [14]. The ESCRT machinery consists of four multiprotein complexes (ESCRT 0, I, II, and III) and accessory proteins (TSG101, ALIX, Vps4, and VTA1). Although ESCRT units are released into the cytosol for recycling, some accessory proteins such as TSG101, HRS, and ALIX remain in exosomes as markers. Alternatively, recent evidence has shown that ESCRT-independent mechanisms exist for exosome formation and release that are based on neutral sphingomyelinase (nSMase)-dependent ceramide formation. This lipid could facilitate membrane invagination of ILVs through its cone-shaped structure [15,16]. Moreover, proteins from the tetraspanin family such as CD9, CD63, CD81, and CD82 are regulators of ESCRT-independent endosomal sorting. For instance, it has been reported that CD63 allows for sorting of a melanosomal protein in ILVs in human melanoma cells [17].

The mechanism involved in the secretion of EVs formed by direct budding from the plasma membrane, such as MVs, is less well characterized and has only started to emerge. Evidence has revealed that recruitment of the ESCRT-I subunits TSG101 and Vps4 to the plasma membrane through binding to a tetrapeptide protein within the Arrestin 1 domain-containing protein 1 (ARRDC1) results in the release of MVs containing TSG101, ARRDC1, and other cellular proteins [18]. Several factors such as redistribution of phospholipids including reposition of phosphatidylserine to the outer leaflet, contraction of actin/myosin machinery, and extracellular concentration of Ca^+2^ can impact MV formation and alter membrane fluidity and deformability [19]. In addition, enzymes that transfer lipids from one leaflet of the plasma membrane to the other such as flippases, floppases, and scramblases play an important role in MV development [20].

EVs are released to the extracellular space due to the presence of Rab proteins, which are essential regulators of intracellular vesicle transport between compartments. Rabs can be involved in vesicle budding, trafficking through interaction with the cytoskeleton, or docking to the membrane of an acceptor compartment [21]. Secreted EVs establish interactions with recipient cells and transmit the information. The interaction can be through ligand/receptor molecules on their respective surfaces mediated by classical adhesion molecules including integrin and intracellular adhesion molecules. Additionally, EVs can deliver their content by endocytic processes such as clathrin- and caveolin-mediated endocytosis, lipid raft endocytosis, phagocytosis, and micropinocytosis, probably guided by vesicle membrane composition and surface molecule profile [22].

### 2.3. Components of EVs

The biochemical composition of EVs has been studied in several populations and likely depends on the mode of biogenesis, cell type, and physiological conditions. Likewise, the isolation methods that are used can influence the composition of EVs. Thus, characterization of all their components remains unclear. Recent technology advances and new methodologies such as proteomics, lipidomics, and transcriptomics have allowed the identification of different types of cargos in EVs (Figure 1). The derived information has been integrated in several databases such as Exocarta [23], Vesiclepedia [24], and EVpedia [25], which serve as repositories and tools that help to decipher vesicle loading and functions. Overall, all EVs are loaded with proteins, lipids, and nucleic acids, and their cargo can be specific to the vesicle and cell type. This section will describe the content of EVs in general terms.

Proteins: The protein cargo of EVs in various cell types has been extensively reviewed [26,27]. Diverse proteomics analyses of exosomes have identified proteins associated with the biogenesis mode, including proteins linked to the endosomal pathway [28]. In fact, components of ESCRT have been found in exosomes, including ALIX, TSG101, and HRS [29]. In addition, proteins involved in the trafficking and release of EVs such as RAB27A, RAB11B, and ARF6 are commonly identified [30]. As mentioned above, EVs are rich in tetraspanin proteins including CD63, CD81, and CD9, which are being used as exosome markers. These proteins can target specific cells, such as endothelial cells, to promote angiogenesis [31]. In addition, several metabolic enzymes such as ATPase, glyceraldehyde-3-phosphate dehydrogenase, enolase-1, and pyruvate kinase type M2 have been detected in EVs by proteomic analysis [32]. Likewise, heat shock proteins including HSP70 and HSP90 and major histocompatibility complex (MHC I and II) are found in EVs derived from most cell types and are involved in antigen binding and presentation [31].

Lipids: The content of lipids in EVs are not fully characterized, but in general it is known that lipid composition shares common features with the origin cells. Outstanding reviews about this topic are available in the literature [33,34]. It has been suggested that some lipids can be specifically associated with different types of EVs, depending on whether they originate from MVB or from plasma membrane [35]. In general, EVs are rich in phosphatidylcholine, phosphatidylserine, phosphatidylethanolamine, phosphatidylinositols, phosphatidic acid, cholesterol, ceramides, sphingomyelin, glycosphingolipids, and other less abundant lipids [36]. Exosomes are rich in phosphatidylserine facing the extracellular milieu, an attribute that likely facilitates their internalization by recipient cells [37]. A single universal marker for MVs is less well defined. However, ARF-6 and CD40 are frequently found in MVs derived from tumor cells [38]. Since EVs are membrane-limited vesicles, their lipid bilayer has a protective function that preserves internal cargo from proteolytic or RNAase degradation.

Nucleic acids: Recent research has demonstrated a varied composition of genetic material in EVs. Diverse studies have found genomic DNA, complementary DNA (cDNA), and transposable elements in extracellular vesicles [39]. Currently, research is focused on RNA content. Extensive scientific evidence shows that EVs carry functional RNAs that play a pivotal role in cell-to-cell communication and regulation of cellular processes. In this sense, EVs contain mRNAs, non-coding RNAs (ncRNAs) including micro-RNAs (miRNAs), small nuclear RNAs (snRNAs), and transfer RNAs (tRNAs) [40]. In particular, miRNAs exported through EVs have a relevant role since they can post-transcriptionally regulate gene expression in distant cells [41,42,43]. In this context, a database called EV miRNAs has been created with data from multiple studies analyzing miRNA expression profiles in various types of EVs from distinct cell types [44]. Since exosomal miRNAs play a pivotal role in several diseases, have great stability, and reach a high concentration in circulation, they have great potential as disease biomarkers for diagnosis or prognosis, and as therapeutic targets [8,45].

## 3. Intercellular Communication by EVs in Intestinal Homeostasis

Intestinal homeostasis depends on complex, dynamic interactions between the microbiota, the epithelium, and the host immune system. Given the complexity of the intestinal ecosystem, regulatory mechanisms involving immune receptors, signaling pathways, regulatory proteins, and miRNAs are required to ensure symbiosis and avoid exacerbated inflammatory responses that could lead to pathological states. In this scenario, intercellular communication is crucial to orchestrate balanced responses to preserve intestinal homeostasis.

There is mounting evidence that intra- and interkingdom crosstalk is mediated by EVs released by gut microbiota or by host intestinal cells. This review is focused on host-derived EVs. It is well known that cargos in EVs confer specific properties and functions. For instance, the presence in EVs of metalloproteinases (MMPs), chemokines, cytokines, growth factors, adhesion molecules, and miRNAs plays a crucial role in processes involving the immune system. Differences in the amount of released EVs and their composition have been observed in health and disease states. High levels of EVs with different content were found in samples from patients with inflammatory bowel disease (IBD), which suggests their contribution to intestinal inflammatory processes [46]. The role of EVs as messengers in cell-to-cell communication inside the body is not limited to the gut environment. As mentioned above, it is now well accepted that circulating EVs can reflect diverse healthy and pathologic states of cells and tissues. Therefore, they are being explored as diagnostic biomarkers or therapy tools in several disorders.

In the following sections, we summarize the functions and contribution of host-derived EVs to intestinal homeostasis and immune cell function, according to the available literature. We focus on epithelial barrier integrity, repair and wound healing of epithelial cells, immunity, and microbiota shaping.

### 3.1. EVs Modulate Epithelial Barrier Integrity

The mucosa surface in the gastrointestinal tract comprises a single layer of intestinal epithelial cells (IECs) that is optimized to efficiently absorb nutrients, water, and electrolytes from food. Moreover, this epithelial layer creates an interface that protects from invading pathogens and prevents the ingress of luminal harmful substances. Disruption of this barrier results in increased intestinal permeability, which facilitates the translocation of antigens and bacteria into the bloodstream. Thus, in some diseases of inflammatory origin such as IBD, loss of intestinal barrier function causes tissue injury by an excessive inflammatory response in the gut wall.

EVs play a crucial role in regulating epithelial barrier integrity. Disruption of the intestinal epithelial barrier results in epithelial injury and subjacent inflammation caused by migration of macrophages and polymorphonuclear cells (PMN) across IECs [47]. Experimental evidence indicates that leukocyte migration can affect tight junction (TJ) proteins in IBD [48]. In fact, loss of several key components of the apical junctional complex (AJC) including occludin, claudins, and zonula occludens (ZO) proteins occurs in locations of inflamed tissue with abundant transmigrating neutrophils [49]. It has been shown that PMN-derived soluble mediators such as EVs contribute to these processes, activating the immune response and specific pathways of inflammation depending on their content [50].

Cargo proteins in EVs influence epithelial barrier integrity. Under inflammatory conditions, PMN-derived EVs contain high amounts of MMP-9, a matrix metalloproteinase that mediates negative effects on IEC integrity. MMP-9 disrupts the epithelial barrier by cleaving proteins involved in intercellular adhesions such as desmoglein-2 (DSG-2), a protein that is crucial for barrier integrity through several mechanisms. DGS-2 is linked to the actin cytoskeleton through interactions with adapter proteins and provides structural support for epithelial monolayers. In addition to its adhesion function, DSG-2 regulates intestinal barrier function via p38 MAPK signaling and is required for proper localization of ZO-1 [51,52]. In IECs, DGS-2 has a barrier function that prevents PMNs migration. In the inflamed epithelium, the MMP-9 enzyme contained in EVs released by PMNs degrades DGS-2, thus enabling PMN trafficking across epithelial layers [51]. Conversely, macrophages recruited to intestinal inflamed sites may contribute to tissue homeostasis, as they release EVs with high content in galectin-3 (Gal-3). In macrophages, Gal-3 export through exosomes is regulated by reactive oxygen species and nicotinamide adenine dinucleotide phosphate (NADPH) oxidase-dependent superoxide production activity [53]. This lectin interacts with and stabilizes DSG-2 at the intercellular adhesion sites, which contributes to IEC barrier integrity [54]. In addition, EVs mediate cross-regulation between activated PMNs and macrophages. It has been shown that PMN-derived EVs are taken up by macrophages, leading to rapid Ca+2 flux and release of TGF-β1. By these mechanisms, PMN-derived EVs regulate the immunomodulatory properties of macrophages [55].

Proinflammatory cytokines cause intestinal barrier disruption. A study conducted in luminal aspirates of IBD patients showed that granulocyte-derived EVs release cytokines such as IL-6, IL-8, and TNF-α. In vitro assays revealed that these EVs are taken up by colonic epithelial cells and activate the expression of IL-8, which in turn promotes migration of a high number of macrophages to the IBD epithelium [56]. In addition, it is known that TNF-α increases epithelial permeability via mislocalization of TJ proteins including ZO-1, claudins, and occludin through the activation of the ERK1/2 signaling pathway [57].

The cellular prion protein (PrPC) is released in exosomes from activated platelets that participate directly in cell-to-cell transmission [58]. PrPC is located in cell-to-cell junctions and interacts with desmosome proteins in the intestinal epithelium. Indeed, mice deficient in PrP have greater paracellular permeability than wild-type mice. Alterations in barrier function were also confirmed in in vitro cultures of PrPc knockdown colonic Caco-2 cell monolayers. PrPC deficiency results in decreased levels of E-cadherin, desmoplakin, plakoglobin, claudin-4, occludin, ZO-1, and tricellulin at intercellular contacts in colonic cell monolayers [59]. Interestingly, PrPC localization is altered in colonic epithelia from patients with IBD.

miRNAs in EVs influence epithelial barrier integrity. EVs can also transport miRNAs, which are small non-coding RNAs that modify post-transcriptionally the expression of a huge number of proteins in target cells. These regulatory RNAs bind to complementary sequences at the 3′-untraslated region (UTR) of target mRNAs and interfere with protein synthesis by triggering mRNA degradation or blocking its translation. Several miRNAs have been involved in the regulation of AJC and epithelial barrier dysfunction in intestinal inflammation diseases, such as Crohn’s disease (CD) and ulcerative colitis (UC) [60,61]. In blood, the presence of these miRNAs correlates with their expression changes in the inflamed gut mucosa. Circulating miRNAs are mainly secreted through exosomes, although some reports show that they can also be distributed in association with the RNA-binding protein argonaute-2 or high density lipoproteins (HDL) [62,63]. Here, we consider miRNAs known to be secreted through EVs.

One miRNA deregulated in IBD is miR-21. Increased levels of this miRNA have been found in samples of IBD patients [64,65]. During colonic inflammation, activation of the neuropeptide Substance P signaling pathway triggers miR-21 sorting through exosomes released by colonic epithelial cells [66]. In vitro studies in human colonic cell lines have indicated that miR-21 has a destructive effect on the intestinal barrier, regulating TJs’ permeability through the PTEN/PI3K/Akt signaling pathway. In epithelial cell monolayers, overexpression of this miRNA downregulates occludin and E-cadherin but increases production of prostaglandin E2, IL-6, and IL-8 [67]. Studies performed in an in vivo intestinal barrier dysfunction model induced by ischemia reperfusion confirmed the correlation between upregulation of miR-21 and loss of barrier integrity. Consistently, levels of the TJ proteins occludin and claudin-1 were reduced in the intestinal tissue of treated animals. In this model of barrier disruption, production of IL-6, TNF-α, intercellular adhesion molecule-1 (ICAM-1), and myeloperoxidase (MPO) was also increased [68]. In experimental colitis, miR-21 was shown to control intestinal barrier function by regulating the expression of Ras-related small GTP-binding protein B (RhoB) and cell division control protein 42 (CDC42). Thereby, attenuated expression of miR-21 in gut could prevent barrier disruption and the progression of subjacent inflammatory process [69].

Another miRNA upregulated in IBD is miR-223. Serum levels of miR-223 are higher in CD and UC patients than in healthy controls [70,71]. The IL-23 pathway is important for the onset of IBD since it causes downregulation of claudin-8, an important protein that conforms the structure of intestinal TJs. In a 2,4,6-trinitrobenzene sulfonic acid (TNBS)-induced colitis model in mice, miR-223 was identified as a mediator of the IL-23 pathway that suppresses the activity of claudin-8. Treatment of colitic mice with antagomir-223 restored the levels of claudin-8 and improved the clinical symptoms of sick mice [72]. Mast cells play a critical role in development of IBD. A recent study showed that exosomes from human mast cells are loaded with miR-223, and that delivery of this miRNA into IECs downregulates the expression of the TJ-related proteins ZO-1, occludin, and claudin-8, which in turn elicit the subsequent increase in intestinal permeability that contributes to inflammation and disease development [73].

High levels of miR-29a, miR-122, and miR-874 have also been found in blood, small bowel, and colon tissues of IBD patients and other pathological conditions associated with increased intestinal permeability. Although their secretion through EVs has not specifically been investigated in IBD, all these miRNAs have been found deregulated in blood circulating exosomes from colorectal cancer patients [74,75,76]. These miRNAs modulate intestinal epithelial barrier through several mechanisms by targeting TJ proteins and key regulatory factors. Concerning miR-29a, one of the mechanisms is downregulation of glutamine synthetase [77]. In gut samples of IBD patients, overexpression of miRNA-29a also reduces the expression of the TJ protein claudin-1 and the transcriptional silencer protein nuclear factor-κB-repressing factor (NκRF). This leads, respectively, to epithelial barrier disruption and highly deregulated transcriptional activity of proinflammatory mediators [78]. Another cellular process regulated by miR-29a is apoptosis. Intestinal epithelial apoptosis is a common histopathological feature of inflammation-related diseases. In UC patients and UC experimental models, miRNA-29 triggers apoptosis of IECs by downregulating anti-apoptotic protein Mcl-1 of the BCL-2 family [79]. Remarkably, miR-29a is also exported through DC-derived EVs [80]. In fact, miR-29 participates in immunity as a key player in the interaction of DCs with T cells. In this case, the targets are molecules involved in antigen presentation and release of cytokines [81].

MiR-122a was also detected in clinical specimens of IBD. This miRNA modulates signaling pathways that control intestinal permeability by targeting epidermal growth factor receptor (EGFR). Downregulation of EGFR leads to increased levels of zonulin, a modulator of intestinal epithelial TJs that increases intestinal permeability [82]. In addition, miR-122 binds to occludin mRNA, promoting its degradation and the subsequent depletion of occludin, which in turn destabilizes the epithelial barrier [83].

Overexpression of miR-874 in ischemic injury has damaging effects on barrier integrity leading to subsequent increased intestinal permeability. This miRNA downregulates aquaporin 3, a protein expressed in gut epithelia with an important role in the integration of TJ complex structure. In this pathological model, high levels of miR-874 also correlated with reduced levels of mucin-2 and the TJ proteins occludin and claudin-1 [84].

### 3.2. EVs Are Involved in Tissue Repair, Mucosal Healing, and Cell Proliferation

In mucosal wounds, EVs from IECs and immune cells can locally modify the expression of growth and transcription factors to control cell migration, proliferation, and differentiation, promoting epithelial healing and tissue repair [85]. Upon injury, a succession of events occurs in epithelial mucosa to bring about wound closure. These involve recruitment of immune cells and release of proteins and mediator molecules. Annexin A1 (ANXA1) is a potent endogenous pro-resolving mediator that facilitates resolution of inflammation and orchestrates wound repair through the formyl-peptide receptor-like pathway. It has been shown that ANXA1-deficient animals exhibit increased susceptibility to dextran sulphate sodium (DSS)-induced colitis with greater clinical morbidity and histopathologic mucosal injury [86]. IEC-derived exosomes contain ANXA1 that facilitates tissue repair by binding to formyl peptide receptors (FPRs) in responsive cells. Activation of this pathway leads to generation of ROS by the epithelial NADPH oxidase NOX1, which in turn controls the activation of focal adhesion mediators [87]. Release of ANXA1-containing EVs increases during wound closure and in IBD patients with acute inflammation and injury. In an experimental in vivo model, the application of exogenous ANXA1 mimetic peptide (Ac2-26) encapsulated in polymeric nanoparticles enhanced healing of murine colonic wounds and improved recovery from induced colitis [88]. Likewise, PMN-derived extracellular vesicles containing ANXA1 mediate anti-inflammatory effects by preventing PMN adhesion to endothelial cells and inhibiting recruitment of PMN to inflamed tissue. Overall, these activities limit progression of the inflammatory process and help its resolution [89].

EVs isolated from the mucosal–luminal interface of IBD patients also contain high levels of the enzyme MPO [90]. Whereas ANXA1 participates in colonic repair, MPO generates reactive oxidants. This activity is beneficial for killing invading pathogens, but in the case of unsuitable or excessive stimulation, it contributes to acute and chronic inflammation [91]. In this context, PMN infiltration of the intestinal mucosa frequently results in severe epithelial injury. Microvesicles released by these immune cells contain highly active MPO, which upon delivery to IECs inhibits wound closure by impairing IEC migration and proliferation [92]. In addition, MPO associated with PMN-derived EVs enhance the inflammatory response through activation of IL-6, IL-8, and MCP-1 secretion by IECs, which facilitates leukocyte recruitment and triggers the upregulation of adhesion molecules. Besides proinflammatory EVs that help other immune cells to reach damaged, inflamed sites, neutrophils secrete EVs with anti-inflammatory functions to limit the excessive immune responses of neighboring cells such as macrophages, monocytes, and natural killer (NK) cells [93]. The impact of neutrophil microvesicles loaded with MPO on other cell types has also been reported. Exposure of human umbilical vein endothelial cells to these EVs results in disturbances in membrane integrity and morphological changes [94]. Moreover, M2 macrophages are involved in the resolution of inflammation and intestinal repair. These immune cells promote proliferation of colonic epithelial cells and wound healing in an exosome-dependent manner, which involves miR-590-3p. This miRNA activates transcription of YAP-regulated gens by targeting the serine/threonine protein kinase LATS1. In addition, miR-590-3p blocks activation of pro-inflammatory cytokines such as TNF-α, IL-1β, and IL-6 [95].

Other immune cells release EVs with wound healing activity on endothelial cells. In a diabetic rat model, macrophage-derived EVs were shown to accelerate re-epithelialization of diabetic lesions. The mechanism is related with their anti-inflammatory effects, which diminish the production of proinflammatory cytokines (IL-6 and TNF-α) and enzymes (MMP-9). TGF-β is one of the mediators secreted by macrophages as part of the inflammatory response. Regardless of its role in immune function, TGF-β also participates in tissue repair. In the presence of this cytokine, fibroblasts in damaged inflamed lesions undergo phenotypic differentiation to myofibroblasts, which contributes to wound healing [96]. In the same way, exosome-containing TGF-β released by injured epithelial cells activate fibroblasts to initiate tissue regeneration responses. Indeed, these exosomes trigger cell proliferation together with production of type I collagen and expression of F-actin by fibroblasts. The resulting fibrosis and increased matrix deposition at the injury site contribute to wound healing [97].

Fibroblasts promote epithelial cell motility through the cell surface tetraspanin CD81. In breast cancer, fibroblast-derived EVs contain CD81 and trigger signaling events to confer cancer cells’ invasive behavior [98]. The Wnt signaling pathway has a relevant role in the regulation of intestinal epithelial cell proliferation [99]. In fact, WNT/β-catenin is activated to maintain homeostasis and achieve wound closure in intestinal epithelium [100]. Some components of this pathway can be secreted into exosomes from gut epithelial and immune cells. For instance, Wnt5b is secreted within exosomes derived from Caco-2 cells. The Wnt5b-associated exosomes were shown to activate cell migration and proliferation of other cancer cells in a paracrine manner [101]. Similarly, macrophages generate exosomes containing Wnt5b that are crucial for the regeneration response in the gut. Following radiation injury, these exosomes activate enterocyte survival through the WNT pathway [102].

Another essential protein controlling IEC migration and proliferation is glycoprotein A33 (GPA33). This is a specific cell surface marker with properties in cell-to-cell communication, including cell adhesion, trafficking, and immune response. GPA33 is expressed in normal stomach, small intestine, colon, and rectal epithelial cells, and in almost all colon cancer. For this reason, it has been proposed as a potential biomarker for diagnosis and prognosis of colorectal cancer [103]. There is evidence that GPA33 modulates intestinal barrier integrity. GPA33 knockout mice show impaired epithelial barrier function, with quick onset and delayed resolution of DSS-induced colitis. In addition, in this experimental model of colitis, GAP33 deficiency correlates with an increased number of colitis-associated tumors following treatment with the colon-specific mutagen azoxymethane [104]. Studies using the 2,4,6-trinitrobenzene sulfonic acid/ethanol-induced colitis model confirmed the role of GPA33. In this experimental model, mice deficient in GPA33 also showed impaired ability to resolve tissue damage due to delayed epithelial cell proliferation and weakened adaptative immunity. Consistently, GPA33-deficient mice showed higher mortality than wild-type mice following colitis induction [105].

### 3.3. EVs Orchestrate Regulation of Intestinal Immunity

#### 3.3.1. IEC-Derived EVs

**Figure 2:**
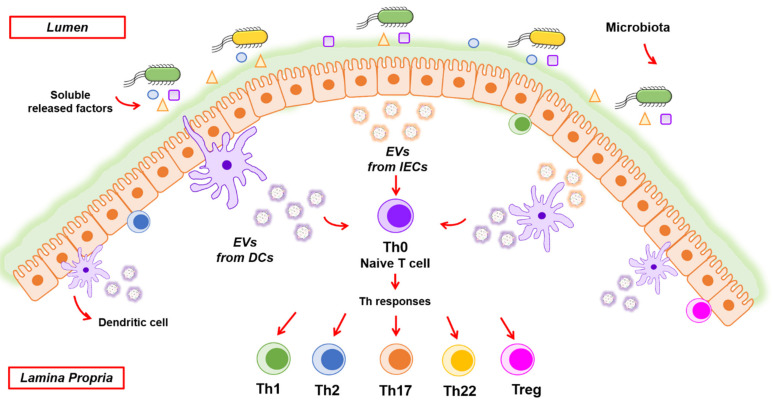
Modulation of immune responses by EVs originated from intestinal epithelial and immune cells. Schematic view of the intestinal mucosa showing the epithelium and the underlying immune system. These host cells receive information from microbiota mainly through secreted factors and lumen antigens that can diffuse through the mucus layer and initiate appropriate immune responses. In this scenario, EVs are key for the host to communicate with neighboring cells. Intestinal epithelial cells (IECs) release EVs from the basolateral side (colored in brown) that participate in the crosstalk with lymphocytes and dendritic cells, being able to activate naïve T cells towards immunogenic (Th) or tolerogenic (Treg) responses depending on the exosome-expressed epitopes and cargo. DCs are the main antigen-presenting cells in the gut lamina propria. These immune cells can integrate information either directly from the intestinal lumen or transmitted through EVs secreted by other cell types under healthy and pathological conditions (intestinal infections, inflammation, or cancer). Once activated, DCs secrete EVs (colored in purple) containing MHC and costimulatory molecules that mediate antigen presentation and immunomodulatory effects towards CD4+ or CD8+ T cells, eliciting suitable T cell responses.

IECs release vesicles from the apical and basolateral sides. Mounting evidence indicates that IEC-derived EVs serve as crucial messengers for intercellular communication in the gut. These vesicles transport diverse molecules that participate in crosstalk with the immune system to maintain intestinal homeostasis (Figure 2). This activity is at least in part conferred by mediators that are expressed on the surface of EVs, such as molecules involved in the antigen presentation function. Although IECs are not specialized antigen-presenting cells (APC), an analysis of samples from small intestinal biopsies showed that IECs constitutively express MHC I and MHC II, HLA-DM, CD63, CD68, A33, and other molecules typically found in exosomes derived from professional APCs. These molecules confer antigen sampling and processing capacities on IECs. Their intracellular distribution and trafficking points to their secretion through exosomes released from the basolateral compartment of the normal gut epithelium [106]. Secretion of immune molecules involved in antigen presentation by IEC-derived EVs was also evidenced in human and murine IEC lines. In the human HT-29 and T-84 cell lines, the expression of MHC I and MHC II and the release of EVs containing these immune mediators was shown to be upregulated by inflammatory signals [107]. In the murine epithelial cell line MODE K, released exosomes also display MHC I/II peptides and other immune markers such as CD9, CD81, CD82, and A33 antigen and were able to activate immunogenic or tolerogenic immune responses depending on the exosome-expressed epitopes [108]. Thus, IEC-derived exosomes may influence antigen presentation in the intestinal mucosa independently of direct interaction with the effector immune cells. In this context, exosomes derived from the intestinal epithelial cell line T-84 have been shown to establish communication with DCs, which in turn elicit T-cell activation. Whereas direct presentation of IEC-derived exosomes to lymphocytes is ineffective in driving appropriate responses, exosome interaction with DCs produces stronger peptide presentation to T cells [109]. Taken together, these studies indicate that EVs released from the basolateral side of enterocytes are independent antigen-carrying entities that connect detection of luminal antigens with the local immune system [110].

IEC-derived EVs can also drive tolerogenic responses in the gut. This activity has been associated with integrin αvβ6, whose expression is upregulated in IECs in response to luminal antigens. Remarkably, exosomes released by IECs act as carriers of food and luminal antigens. Studies performed in both mice and intestinal epithelial cell lines challenged to ovalbumin as a food antigen revealed that IEC-derived exosomes loaded with αvβ6 and OVA could prime DCs to produce a high amount of active TGF-β and promote generation of regulatory T (Treg) cells. In contrast, DCs challenged directly with OVA in the absence of IEC-derived EVs express high levels of the inactive form of TGF-β, known as latent TGF-β. In fact, integrin αvβ6 delivered by IEC-derived EVs plays an important role in the conversion of latent TGF-β into the active form in DCs. Moreover, these exosomes inhibit in vivo Th2 immune responses towards food antigens. These findings proved that IEC-derived exosomes carrying αvβ6 and food antigens are part of intestinal immune mechanisms involved in the development of tolerance to foreign antigens [111].

Under physiological conditions, IEC-derived EVs are also loaded with TGF-β1. In a mice model of IBD, administration of these vesicles ameliorates inflammation by inducing immunosuppressive DCs and Treg cells, whereas inhibition of EV production in vivo aggravates IBD. These IEC-derived EVs mainly localize with the epithelial cell adhesion molecule (EpCAM) within the intestinal tract. This association is relevant, since the protective effects of TGF-β1-rich EVs are dampened in EpCAM knockout mice. In addition, the severity of IBD was higher in EpCAM-deficient animals than in wild-type mice [112].

Conversely, when EVs are released by injured epithelial cells they promote immune dysfunction. It has been shown that when trauma and hemorrhagic shock occurs, rat gut epithelial cell-derived exosomes alter the maturation and function of DCs by increasing cell apoptosis and decreasing the expression of CD80 and CD86 surface markers and their capacity to drive T-cell responses through antigen presentation [113].

#### 3.3.2. EVs Derived from Immune Cells

DCs are recognized as the main antigen-presenting cells in the gut lamina propria and can integrate signals from the intestinal lumen and shape appropriate immune responses. In addition to direct sampling of luminal microbes, DCs receive information from EVs released by other cell types and/or tissues under healthy and pathological conditions that include intestinal infections, inflammation, or cancer. IECs are key players in this indirect pathway of communication, since once activated by an external stimulus they transmit the information to the underlying immune cells by releasing EVs and soluble mediators (Figure 2). Then, activated DCs elicit suitable T-cell responses through antigen presentation and secretion of specific cytokines. In this context, activated DCs secrete EVs containing MHC class I and class II complexes and costimulatory molecules that contribute to antigen presentation and immunomodulatory effects towards CD4+ or CD8+ T cells.

Initial studies on DC-derived EVs showed that injection of DC-derived exosomes to mice induced antigen-specific naïve CD4+ T-cell activation, whereas in in vitro models of cell culture only exosomes derived from mature DCs expressing CD80 and CD86 costimulatory markers could trigger appropriate naïve CD4+ activation. In fact, these exosomes allow the exchange of functional MHC complexes between DCs, which provides a mechanism to magnify adaptive immune responses [114]. Similarly, exosomes derived from DCs challenged with lipopolysaccharide (LPS), but not exosomes from immature DCs, were shown to be loaded with MHC class II, B7.2, and intercellular adhesion molecule 1 (ICAM-1). Functional studies also revealed that the presence of these immune molecules in the exosomes is required for their capacity to prime naive T cells [115]. Like other cell types, human DCs produce different kinds of EVs. The proteomic analysis of the heterogeneous EVs population showed that some molecular markers, such as MHC-I/II, flotillin, and heat shock 70-kDa proteins, are similarly present in all EVs, whereas other proteins are mainly associated with a particular vesicle subtype. For instance, exosomes are enriched in tetraspanins CD9, CD63, and CD81 compared to other extracellular vesicles. In fact, these molecules have been proposed as exosome surface markers for immune-based separation methods [31,116]. Functional studies to characterize the effect of EVs isolated from primary DC cultures revealed that both large EVs and small EVs secreted from mature DCs could induce CD4+ T cell activation in vitro. In contrast, vesicles secreted by immature DCs differed in T-helper-driven responses. Large EVs promoted secretion of Th2 cytokines whereas small EVs prompted Th1 cytokine secretion. Nevertheless, these differences were no longer observed following DC maturation, and all EVs were able to induce INF-γ and the subsequent Th1 response [117].

Besides DCs, other immune cells participate in the maintenance of intestinal homeostasis, and their regulation is crucial for controlling inflammation and IBD pathogenesis. For instance, EVs from granulocytic myeloid-derived suppressors cells (G-MDSC) attenuated DSS-induced colitis in mice. Treated animals showed reduced infiltration of inflammatory cells in the damaged mucosa. Moreover, treatment with G-MDSC-derived exosomes results in increased production of Treg cells in mesenteric lymph nodes together with decreased amount of Th1 cells, and lower serum levels of the proinflammatory cytokines TNF-α and INF-γ. These effects were associated with arginase-1 contained in derived exosomes [118]. Wong et al. (2016) stimulated macrophages RAW264.7 with exosomes isolated from serum of DSS-treated colitic mice to analyze how circulating exosomes may contribute to the pathogenesis of IBD. Comparison with stimulations performed with serum exosomes from control mice revealed that exosomes from colitic mice activated proinflammatory signaling cascades and triggered upregulation of TNF-α production [119]. A proteomic analysis identified 56 differentially expressed proteins in exosomes from colitic mice, most of them related with processes involved in macrophage activation, such as the complement system and the coagulation pathway [119].

The role of PMN cells in tissue repair and leukocyte trafficking during inflammation is well-established. They release potent antimicrobial agents that cause degranulation. Simultaneously, PMNs release cell surface-derived microvesicles with different functions in cell-to-cell communication. PMN-derived EVs modulate the maturation and function of macrophages and DCs during inflammatory processes. Stimulation of immature DCs with PMN-derived EVs results in cell morphology changes, reduction of DC phagocytic activity, and increased TGF-β1 release. In addition, PMN-derived EVs partially inhibit the maturation of DCs exposed to LPS. Under these conditions, activated DCs showed decreased secretion of inflammatory cytokines such as IL-8 and IL-6, as well as reduced capacity to induce T-cell proliferation [120]. A lipidomic study performed in phagocytes revealed that the lipid mediator profile of the released EVs differs between cell types and maturation stages. The study showed specific regulation of PMN-derived EV production by lipid mediators during the stages of the inflammatory process, which determine rapid resolution of inflammation [121]. Macrophage-derived EVs carry leukotrienes, which are potent inflammatory mediators participating in granulocyte recruitment. The expression of leukotriene biosynthesis enzymes in macrophages and subsequent leukotriene exosome release are upregulated by TGF-β1 [122].

Besides immunomodulatory effects, EVs released by infiltrating PMN mediate pathological effects in the intestinal mucosa. This is particularly relevant in IBD. These EVs can cause genotoxicity with clear implications for cancer development. PMN-induced genotoxicity has been attributed to oxidative stress generated by the release of reactive oxygen species. Recent evidence has indicated that PMN-derived EVs can also contribute to these effects by a different mechanism. PMN-derived EVs carry the proinflammatory miRNAs miR-23a and miR-155, which, upon intracellular delivery to IECs, promote double-strand breaks in DNA by targeting key regulators of the homologous recombination repair system. The increase in such lesions in damaged epithelium leads to impaired mucosa healing and genomic instability that in turn accelerate the progression of colorectal cancer [123].

#### 3.3.3. EVs Derived from Mesenchymal Stem Cells

Recent studies show the therapeutic potential of mesenchymal stem cells (MSCs) against autoimmune and inflammatory diseases. The capacity of MSCs to modulate phenotype and function of immune cells largely depends on secreted EVs [124]. Several reports indicated that administration of EVs derived from MSCs isolated from different sources that include bone marrow, umbilical cord, or olfactory lamina propria ameliorate experimental colitis in mice [125,126,127,128,129,130,131]. In all these models, animals treated with MCS-derived EVs showed preserved intestinal barrier integrity, reduced infiltration of inflammatory cells in the damaged mucosa, reduced secretion of pro-inflammatory cytokines, and expansion of Treg cells. Several vesicular factors and mechanisms have been shown to mediate these effects. Concerning EVs released by bone marrow derived MSCs, the mechanism involved downregulation of NF-κB p65 and the oxidative stress enzymes iNOS and COX-2 in injured colon. Additionally, these EVs also repressed apoptosis by inhibiting proteolytic cleavage of diverse caspases in colitis [127]. Cao et al. (2019) evidenced that amelioration of colitis by bone marrow MSC-derived EVs is associated with their ability to induce polarization of colon macrophages into the immunosuppressive M2 phenotype. The increase in colonic IL-10 and TGF-β levels allows for repair and regeneration of intestinal epithelial barrier [129]. Among the multiple exosome-containing proteins that could mediate the anticolitic effects, metallothionein-2 was shown to be required to suppress inflammatory responses [126]. Studies performed with human umbilical cord MSCs revealed new mechanisms responsible for the anti-inflammatory effects of the derived exosomes in experimental models of IBD. These mechanisms involve modulation of post-translational modifications of proteins, including ubiquitination and neddylation, thus controlling conformation and stability of target proteins [125,130]. Neddylation refers to the covalent binding of a ubiquitin-like protein to the target proteins by the sequential action of three enzymes in a process that resembles ubiquitination. However, this modification does not target the protein for degradation but promotes conformational changes that impact on protein interactions and activity. Wang et al. (2020) showed that miR-326, which is highly expressed in exosomes derived from umbilical cord MSCs, plays a relevant role in inhibiting neddylation and NF-κB signaling in murine experimental IBD [125].

Intestinal inflammation is closely associated with epithelial barrier disruption. Interestingly, exosomes derived from MSCs contain miR-34a/c-5p and miR-29b-3p, which improve intestinal barrier function by targeting the transcriptional repressor Snail (Table 1). Both miRNAs and several claudins are downregulated in damaged human intestinal tissues. Following exosome uptake, miR-34a/c-5p and miR-29b-3p activate expression of claudin-3, claudin-2, and ZO-1 in IECs [132].

#### 3.3.4. Immune Regulation by EV-Associated miRNAs

As mentioned above, besides lipidic and peptide mediators, EVs also carry regulatory RNAs. By this mechanism, miRNAs can be transferred between cells and mediate target gene repression. In this context, miRNAs modulate gene expression of effector molecules involved in innate and adaptative immune responses. In fact, dysregulation of known master regulators of inflammation and immunity, such as miR-155 and miR-146, is a general trait in IBD [133]. Both miRNAs are released from DCs within exosomes and are transferred between immune cells in vivo [134].

In IBD patients with colorectal cancer, overexpression of miR-155 has been detected in neoplastic and non-neoplastic mucosa sections, which suggests a connection between dysregulation of this miRNA and the risk of IBD patients to develop colorectal cancer. Upregulation of miR-155, and of miR-21, promotes microsatellite instability by targeting the main proteins involved in the mismatch repair pathway [135]. Acute experimental models of colitis using miR-155-deficient animals confirmed the role of miR-155 in assisting proinflammatory responses in IBD. Relative to wild-type mice, deficiency in miR-155 weakened the severity of colitis by altering inflammatory cellular responses. Remarkably, knockout mice had decreased production of local and systemic inflammatory cytokines, and reduced numbers of CD4+ T cells involved in Th1 and Th17 responses [136]. Other studies were conducted to identify the targets regulated by miR-155 in IBD models. In IBD, intestinal myofibroblasts greatly contribute to mucosal damage. Pathak et al. (2015) investigated the role of miR-155 in inflamed myofibroblasts and its modulation in UC. The study showed that miR-155 is upregulated in myofibroblasts from biopsies of UC patients compared to healthy control samples. In intestinal myofibroblasts isolated from control subjects, expression of miR-155 is increased by treatment with proinflammatory mediators such as TNF-α and LPS, whereas TGF-β1 does not modify miRNA expression [137]. In this model, the suppressor of cytokine signaling 1 (SOCS-1) was identified as the main direct miR-155 target responsible for the inflammatory effects. SOCS1 is a proven feedback inhibitor of inflammation, and its downregulation by miR-155 leads to increased production of inflammatory cytokines such as IL-6 and IL-8. This study revealed the mechanism involving miR-155 in UC progression. Inflammatory mediators induce miR-155 expression in intestinal myofibroblasts of UC patients. In turn, this miRNA downregulates the expression of SOCS1, which supports the inflammatory phenotype [137]. SOCS-1 is not the only miR-155 target associated with the IBD phenotype. In vitro studies performed in macrophage cell lines such as Raw264.7 and primary bone marrow-derived macrophages have shown that miR-155 effects on proliferation and release of proinflammatory cytokines are associated with downregulation of the SH2-containing inositol 5′-polyphosphatase (SHIP-1), an important negative regulator of the PI3K-Akt signaling pathway. The role of SHIP-1 as mediator of miR-155 inflammatory effects was confirmed in experimental models of colitis. Colitic mice administered with antagomir-155 showed increased SHIP-1 expression and reduced intestinal inflammation [138]. Taken together, available experimental evidence points to miR-155 as a potential therapy target in IBD.

Another crucial actor in immune regulation is miRNA-146, which post-transcriptionally regulates the expression of toll-like receptors and downstream molecules of their signaling pathways in different subtypes of intestinal epithelial and immune cells. Whereas miR-155 contributes to colitis and mucosal damage, miR-146a suppresses inflammation in experimental models of inflammation. A study performed in an endotoxin-induced inflammation model in mice provided evidence that miRNAs exported through exosomes undergo functional transfer between cells and regulate the inflammatory response. In this in vivo model of inflammation, the administration of exosomes carrying miR-146a reduced TNF-α and IL-6 serum levels compared to mice that received miR-146a-deficient exosomes. In addition, the amount of miR-146a found in tissues such as bone marrow, liver, and spleen, was higher in mice that received exosomes containing miR-146a. Consequently, the expression of miR-146a targets such as IRAK-1 and TRAF-6 was repressed in the tissues of the treated animals [134]. The miR-146 family includes miR-146b, which originated from a different chromosome, and as its counterpart mediates anti-inflammatory effects in the intestine. In the DSS-induced colitis model, administration of miR-146b intraperitoneally enhances epithelial barrier function, relieves intestinal inflammation, and improves the survival rate of treated mice. These effects depend on the NF-κB activation that follows miR-146b-mediated downregulation of Siah2, a protein involved in the ubiquitination of proteins involved in signal transduction from several TNF receptors [139]. Another feature attributed to miR-146a is its protective role against ischemia/reperfusion injury in small intestine. Plasma and intestinal tissue samples from patients with mesenteric ischemia showed lower miR-146a expression levels than controls. Downregulation of miR-146a correlated with upregulation of its targets TLR4, TRAF6, and nuclear NF-κB p65, and with high levels of activated caspase-3. Conversely, upregulation of miR-146a in rat IECs caused an increase in survival and decreased cell apoptosis, together with downregulation of its targets. These findings prove the role of miR-146a in improving intestinal epithelial cell survival in ischemia reperfusion injury [140].

As stated above, upregulation of miR-223 in IBD is related with its secretion through mast cell-derived exosomes. Besides its role in controlling barrier integrity, miR-223 activates NF-κβ signaling and production of proinflammatory cytokines, which exacerbates the progress of IBD. This miRNA suppresses IκBα levels (NF-κβ inhibitor) through downregulation of its target FOXO3a [141]. Although these findings indicate a positive correlation between upregulation of miR-223 and IBD development, its exact role in intestinal inflammation is controversial since deficiency of miR-223 has been related to high susceptibility to develop induced colitis in mice. The miR-223-deficient mice exhibited reduced levels of conventional DCs and macrophages that express the chemokine CX3CXR. These macrophages are present throughout the intestinal tract and display high phagocytic activity, which protects the host against infections [142]. Moreover, miR-223 has been shown to limit intestinal inflammation by targeting NLRP3 inflammasome expression in monocytes to inhibit IL-1β production during murine colitis. Reduced IL-1β levels result in amelioration of injury and improvement of clinical and histological signs of colitis [143]. These controversial findings point to a role of miR-223 in the control of intestinal homeostasis by modulating innate immune responses during inflammation.

Other less described miRNAs such as miR-16 and miR-10a could be important in the physiological control of inflammation. These miRNAs have been found as exosomal circulating miRNAs in serum of healthy mice. These exosomes were shown to be originated from different tissues including the intestine [144,145]. In humans, miR-16 is highly expressed in UC [146]. The effects of this miRNA may be attributed, at least partially, to downregulation of its target adenosine A2a receptor (A2aAR), which is essential for adenosine-mediated inhibition of the NF-κB signaling pathway during inflammation. Transfection of HT-29 cells with miR-16 mimics confirmed translocation of NF-κβ p65 into the nucleus and the subsequent release of inflammatory cytokines. In contrast, treatment with an miR-16 inhibitor counteracted miR-16-derived effects [147]. Some reports point to miR-10a as another of the miRNA regulating immune pathways in the intestinal environment that could play a role in the pathogenesis and progression of IBD. This miRNA has been found in lower amounts in samples from patients with IBD. Consequently, miR-10 targets such as NOD2 and IL-12/IL-23p40 were upregulated in IBD patients compared to controls. Treatment with anti-TNF-α antibodies markedly increases the expression of miR-10 and downregulates the expression of NOD2 and IL-12/IL-23p40 in inflamed mucosa. Besides these effects, miR-10a inhibits DC activation and suppresses Th1 and Th17 cell immune responses [148]. Similar findings were observed in mice with colitis. These animals expressed higher levels of IL-12/IL-23p40 and lower levels of intestinal miR-10a than control mice. Interestingly, expression of miR-10a is downregulated by gut microbiota through the MyD88-dependent pathway, providing in this way a mechanism that contributes to the maintenance of intestinal homeostasis [149].

Treg cells have been shown to exert beneficial effects on IBD through a mechanism that depends on miR-195a-3p secreted through exosomes. In fact, this miRNA is highly expressed in Treg-derived exosomes. Liao et al. (2020) showed that Treg exosomes alleviate the severity of IBD in mice challenged with dextran sodium sulphate by transferring miR-195a-3p into IECs. This miRNA inhibits apoptosis by targeting caspase-12, allowing cell proliferation and repair of the intestinal epithelial barrier [150].

Overall, these findings indicate that host-derived EVs can influence intestinal homeostasis and orchestrate immune responses in different T cell phenotypes. Since the molecules delivered through EVs differ depending on external stimuli or pathological status, the derived response can be positive or negative, protecting or injuring the host. Scientific evidence in this field is increasing and greatly contributes to the understanding of exosome mechanisms, which may lead to exosome-based approaches for IBD therapy.

### 3.4. EVs Released by Cells of the Intestinal Mucosa Shape and Modulate Gut Bacteria

#### 3.4.1. Control of Enteric Pathogens

Recent evidence has suggested that IEC-derived EVs could confer protection against enteropathogenic infections, helping barrier integrity restoration and pathogen clearance. In the context of intestinal infection, the parasite *Cryptosporidium parvum* activates production and luminal secretion of exosomes by infected IECs. The mechanism involves TLR-4 signaling, which promotes the SNAP23-associated vesicular exocytotic process. In addition, exosomes are loaded with antimicrobial peptides of epithelial cell origin, including cathelicidin-37 and β-defensin-2. Thereby, exposure of sporozoites from *Cryptosporidium parvum* to IEC-derived exosomes decreases their infectivity in vitro and ex vivo, which suggests exosome roles in antimicrobial defense against invading pathogens within the intestinal mucosa [151].

In addition to exosomes from IECs, DCs also release exosomes with protective, beneficial properties to the host. There is evidence that *Schistosoma japonicum* is a parasite with protective effects against colitis in mice. Although parasite-based therapy has been suggested as a potential strategy, its effects can be harmful to the host. To overcome this limitation, administration of exosomes from DCs stimulated with soluble egg antigen from *Schistosoma japonicum* was evaluated as an alternative treatment in the DSS-induced colitis model in mice. The study revealed that exosome-based treatment improves disease and histological scores, which suggests its potential use as a new therapy tool in IBD [152].

#### 3.4.2. Micro-RNAs Transported through IEC-Derived EV Modulate the Gut Microbiota

The human gastrointestinal tract is colonized by a diverse microbial community known as the gut microbiota, which is essential to intestinal homeostasis and human health. In addition to its contribution to food digestion and nutrient metabolism, the gut microbiota plays a fundamental role in host immune system development and in the modulation of gut barrier and immune responses [153]. Studies on gut microbiota have increased exponentially in the last few years. From these studies, we have learned that imbalances in microbiota composition and diversity (dysbiosis) disturb host balanced responses and contribute to a wide variety of inflammatory, autoimmune, metabolic, and neurological diseases [154,155,156]. For instance, there is evidence that microbiota composition is altered in IBD patients and that bacterial translocation to blood occurs recurrently [157]. To preserve intestinal homeostasis in such a densely populated environment, elaborated regulatory networks are required to ensure symbiosis and avoid aberrant responses. Regulation by miRNAs is among these mechanisms with a relevant role in inter-kingdom communication. Many studies indicate that microbiota and host miRNAs regulate each other. Gut bacteria have a great impact on miRNA expression, and host miRNAs shape and regulate gut microbiota [158,159,160]. Remarkably, host micro-RNAs in the intestinal lumen are exported through exosomes, mainly derived from IECs [161]. Currently, the study of fecal miRNAs is receiving great interest for their potential application as disease markers. In this context, Liu et al. (2016) reported that miRNAs identified in the gut lumen and feces of mice and humans are present within EVs, which mainly originate from IECs, goblet cells, and Paneth cells, with little or no contribution of immune cells. In addition, this study revealed for the first time that fecal miRNAs shape the gut microbiome [162]. In fact, in silico analysis revealed that bacterial nucleic acid sequences could be targeted by various human miRNAs through complementary base pairing. The authors showed that miRNAs could enter bacteria, interact with nucleic acids, and specifically regulate bacterial growth and gene expression, although the precise mechanisms governing these effects were not elucidated. They found specific correlations between certain miRNAs and bacterial species. In particular, miR-1226-5p promoted growth of *Escherichia coli*, whereas miR-515-5p stimulated growth of *Fusobacterium nucleatum* [162]. The impact of fecal miRNAs on gut microbiota was evidenced in mice deficient in the endoribonuclease Dicer-dependent miRNA processing enzyme. Mutant mice unable to produce miRNAs by IECs display uncontrolled gut microbiota and aggravated DSS-induced colitis. These effects were counteracted by fecal transplantation of miRNAs from wild-type mice [162]. Studies performed in germ-free mice confirmed the role of fecal miRNAs in shaping gut microbiota. In this model too, the amount of fecal miRNA was inversely correlated with the abundance of microbes [163]. In addition, this study revealed that depletion of the gut microbiota by antibiotic treatment also influences fecal miRNA expression, which in turn correlates with the abundance of specific phyla. Authors found a correlation of miR-141-3p with abundance of the phyla Bacteroidetes and Firmicutes, and miR-200a-3p with the phyla Actinobacteria, Bacteroidetes, Cyanobacteria, Firmicutes, and Proteobacteria. All these findings reveal a hitherto unknown route by which the gut microbiome is modulated by the host through miRNAs delivered within EVs released by IECs (Table 1). Moreover, this knowledge suggests that miRNAs might be used as a therapeutic strategy to manipulate microbiota for the treatment of dysbiosis-associated diseases.

The regulatory mechanisms of miRNAs described in this review are summarized in Table 1.

**Table 1:** Micro-RNAs transported through EV important in the gut environment.

Function	miRNA	Mechanism	Reference
Epithelial barrier integrity	miR-21	Downregulates expression of occludin and E-cadherin Increases prostaglandin E2, IL-8, IL-6, TNF-α, ICAM-1, and MPO	[67,68,69]
	miR-223	Secreted by mast cell-derived exosomes Downregulates claudin-8, ZO-1, and occluding in IECS	[73]
	miR-29a	Downregulates expression of claudin-1 and NκRF Induces apoptosis by targeting Mcl-1	[77,78,79]
	miR-122	Upregulates EGFR and zonulin Downregulates occludin expression	[82,83]
	miR-874	Downregulates aquaporin 3, occludin, claudin-1, and mucin-2	[84]
	miR-34a/c and miR-29b-3p	Secreted by MSC-derived exosomes Improve intestinal barrier function by targeting Snail Activates claudin-3, claudin-2, and ZO-1 expression	[132]
Mucosal wound healing	miR-590-3p	Promotes IEC proliferation and wound healing by targeting LATS1 Inhibits induction of TNF-α, IL-1β, and IL-6	[95]
Regulation of intestinal immunity	miR-326	Secreted by MSC-derived exosomes Alleviates IBD by inhibiting protein neddylation and NF-κB signaling	[125]
	miR-155	Contributes to the pathogenesis of IBD Promotes Th1 and Th17 responses Downregulates SOCS1 and increases IL-6 and IL-8 levels Downregulates SHIP-1, a negative regulator of PI3K-Akt pathway	[133,134,136,137,138]
	miR-146a and miR-146b	Suppress inflammation by NF-κB signaling pathway Downregulates TLRs, IRAK-1, and TRAF-6 Increases IL-10 production	[134,139,140]
	miR-223	Activates NF-κβ signaling and production of proinflammatory cytokines Downregulates NLRP3 inflammasome expression	[141,142,143]
	miR-16	Downregulates adenosine A2a receptor and activates NF-κB pathway	[147]
	miR-10a	Decreased in IBD Its targets, NOD2 and Il-12, are upregulated in IBD Associated with Th1 and Th17 immune responses	[148]
	miR-195a-3p	Highly expressed in T reg-derived exosomes Inhibits apoptosis by targeting caspase-12	[150]
Regulation of gut microbiota	miR-1226-5p	Promotes growth of *Escherichia coli* and *Fusobacterium nucleatum*	[162]
	miR-515-5p	Promotes growth of *Fusobacterium nucleatum*	[162]
	miR-141-3p	Associated with abundance of phyla Bacteroidetes and Firmicutes	[163]
	miR-200a-3p	Associated with abundance of phyla Actinobacteria, Bacteroidetes, Cyanobacteria, Firmicutes, and Proteobacteria	[163]

## 4. Conclusions

**Figure 3:**
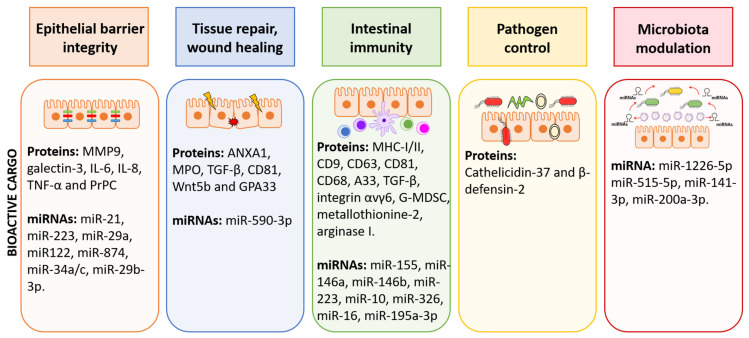
Graphical summary of functions of EVs within the gut environment. EVs derived from IECs and immune cells of the lamina propria contribute to cell-to-cell communication in the gut and have great impact on the homeostasis/inflammation balance. The scheme summarized the bioactive cargo in EVs that can influence epithelial barrier integrity, tissue repair, immune responses, control of pathogens, and microbiota shaping.

Recent knowledge supports the idea that EVs play a relevant role as communication vehicles, specifically transporting and delivering into recipient cells messenger molecules including proteins, lipids, DNA, and RNA. Production of EVs and cargo selection depends on changes in cellular conditions or external stimuli, and consequently, they have a great impact on physiology and body homeostasis. The intestine is critical in controlling human health. Epithelial and immune cells of the intestinal mucosa are constantly exposed to millions of microbes that greatly influence the integrity of intestinal epithelial barrier and immune function. In this ecosystem, both inter-kingdom and intra-host–cell communications are key to control microbiota population and avoid exacerbated inflammatory responses. Host-derived EVs have a relevant role in mediating such interplay, controlling intestinal homeostasis at various levels, as summarized in Figure 3. Little is known about the function of host EVs in controlling gut microbiota, but recent reports point to fecal miRNAs secreted through EVs released by IECs to the gut lumen as modulators of this microbial community. Since genetic and environmental factors including diet, stress, drugs, and dysbiosis affect vesicular cargo, EVs derived from cells of the intestinal mucosa open opportunities to explore their potential as therapeutic targets or diagnostic biomarkers, particularly in IBD.

## Data Availability

Data sharing is not applicable to this article. No new data were created in this review.
